# Identifying anthracnose resistance genes involving in ROS and JA accumulation

**DOI:** 10.1080/15592324.2026.2668219

**Published:** 2026-05-03

**Authors:** Min Zhang, Yuxin Wang, Shuqi Cheng, Weijuan Huang, Yang Liu, Lijing Liu

**Affiliations:** a The Key Laboratory of Plant Development and Environmental Adaptation Biology, Ministry of Education, School of Life Sciences, Shandong University, Qingdao, China

**Keywords:** Anthracnose, ROS, JA, CYS6, BFP1

## Abstract

Anthracnose, caused by one of the top ten most destructive plant fungal pathogen *Colletotrichum* spp., is a devastating disease that infects a wide range of plant species and severely compromises crop yields. Understanding the molecular mechanisms underlying plant resistance to anthracnose is therefore crucial for developing effective control strategies. In previous studies, we have identified two broad-spectrum disease resistance genes, *Cystatin 6* (*CYS6*) and *Botrytis cinerea-induced F-box protein 1* (*BFP1*), which encode proteins modulating reactive oxygen species (ROS) and jasmonic acid (JA) accumulation, respectively. Here, we investigated whether these genes contribute to anthracnose resistance. Our results demonstrated that *Colletotrichum higginsianum* infection caused milder symptoms in *CYS6* and *BFP1* overexpression lines compared to wild-type plants. Importantly, neither *BFP1* nor *CYS6* overexpression adversely affected plant growth or seed production. The evolutionary conservation of CYS6 and BFP1 across different crops were also checked. Our study highlights the potential of *CYS6* and *BFP1* as promising targets for molecular breeding programs aimed at developing anthracnose-resistant crops.

## Introduction

The causative agent of anthracnose, *Colletotrichum* spp., is ranked among the top ten plant fungal pathogens due to its scientific and economic importance.^1^ *Colletotrichum* spp. can infect most plant parts, including roots, stems, leaves, flowers, and seeds, significantly reducing yields in cereal crops, soybeans, tea trees, and fruits.^2-5^ For example, anthracnose can cause up to 100% yield loss in soybean and sorghum and up to 70% yield loss in strawberries.^2^^,^^5^^,^^6^ Additionally, *Colletotrichum* spp. can be cultured in the laboratory and genetically manipulated using molecular technologies, making it a suitable hemi-biotrophic model for studying plant-fungus interactions.^7^ However, the mechanisms plants employ to defend against *Colletotrichum* spp. remain largely unknown.

Pattern-triggered immunity (PTI) is an early plant immune response that confers broad-spectrum disease resistance. It is initiated when membrane-localized pattern recognition receptors (PRRs) recognize pathogen-associated molecular patterns (PAMPs).^8^ Previous studies have demonstrated that PTI is involved in plant defense against anthracnose^9-11^ and that reactive oxygen species (ROS), as signaling molecules, play a crucial role in PTI-mediated broad-spectrum disease resistance.^12^ The accumulation of ROS during PTI is primarily mediated by the NADPH oxidase, Respiratory Burst Oxidase Homolog D (RBOHD). While ROS function as important signaling molecules to activate immune responses, their excessive accumulation can cause cellular damage to plants.^13^ Therefore, both the expression level and activity of RBOHD are tightly regulated during pathogen infection.^14-16^ However, it remains unclear whether elevating the protein stability of RBOHD could enhance plant resistance to anthracnose without incurring fitness costs.

Jasmonic acid (JA) is a phytohormone that mediates plant resistance to multiple pathogens.^17^ Upon pathogen infection, JA level rise rapidly due to the activation of biosynthesis and suppression of catabolism.^18-20^ Previous studies have confirmed that JA plays a critical role in defense against *Colletotrichum* spp.^21-24^ In line with this, it is noteworthy that JA accumulates during *Colletotrichum* spp. infection, and exogenous application of JA can enhance plant resistance to anthracnose.^24-26^ However, while elevating endogenous JA levels can strengthen defense, it often incurs a growth penalty—such as reduced plant size—due to the growth-defense tradeoff.^27^ Therefore, identifying JA-related genes that enhance plant resistance to *Colletotrichum* spp. without compromising fitness remains an important area for further exploration.

*Colletotrichum higginsianum* (*C. higginsianum*) is capable of infecting a wide range of cruciferous species, including the model plant *Arabidopsis.*^28^ Therefore, the *C. higginsianum-Arabidopsis* pathosystem represents an excellent model for investigating the molecular mechanisms and key components underlying plant resistance to anthracnose. In this study, we demonstrated that two previously identified broad-spectrum resistance genes—*Cystatin 6* (*CYS6*) and *Botrytis cinerea-induced F-box protein 1* (*BFP1*), which are involved in ROS and JA accumulation respectively,^16^^,^^18^ also participate in defense responses against *C. higginsianum*. This finding suggests that these pathways may all play critical roles in plant resistance to anthracnose. Given that these genes are evolutionarily conserved across different crops, we propose that they could serve as promising targets for breeding anthracnose resistant cultivars in the future.

## Materials and methods

### Plant materials and growth conditions

*Arabidopsis* WT and transgenic lines were all in the *Col-0* accession in this study. The transgenic lines *CYS6* OE and *BFP1* OE were generated by introducing the *35S_pro_:CYS6-GFP* and *35S_pro_:GFP-BFP1* constructs, respectively, into WT via Agrobacterium-mediated floral dip transformation.^16^^,^^18^ And then T_4_ generation homozygous transgenic lines with stable and uniform expression of the two genes were used for subsequent experiments. Seeds were sterilized with 2.5% (v/v) plant preservative mixture (Coolaber, PTC1000) for 3 d at 4 °C in the dark prior to sowing in soil. Plants were grown in a growth chamber under short-day conditions (12 h light/12 h dark), with a light intensity of 120 µmol m^−2^ s^−1^, a temperature of 22 °C, and a relative humidity of 40%–60%.

### DNA, RNA extraction, and qPCR

To quantify the growth of *C. higginsianum* by qPCR, infected seedlings were collected and ground in liquid nitrogen. Cetyltrimethyl ammonium bromide (CTAB) buffer (2% CTAB, 2% polyvinylpyrrolidone, 100 mM Tris-HCl, pH 8.0, 25 mM ethylenediaminetetraacetic acid, 2 M NaCl) was used to extract DNA. To measure *CYS6* and *BFP1* expression levels in their transgenic lines of *35S*_*pro*_*:CYS6-GFP* and *35S*_*pro*_*:GFP-BFP1*, leaves were harvested from three-week-old plants. Total RNA was extracted using TRIzol Reagent (Takara, 9109). The first-strand cDNA was synthesized by the HiScript III RT SuperMix kit (Vazyme, R323-01). The purified DNA and first-strand cDNA was used for qPCR analysis of *ChTUB* (ChTUB-qF: GAGCGCCCTAACTACGAGAA; ChTUB-qR: CGAAGCAGGACATGGTCATC)*, AtCYS6* (AtCYS6-qF: CTGCAAGCAAGGTGAACATGA; AtCYS6-qR: TCGCCGGTTACCTCAGCT) and *AtBFP1* (AtBFP1-qF: TGTAAACGTGCGGCGGATGTA; AtBFP1-qR: TACAGTGAACTCCACTTGCTCCG) with *AtUBQ5* (AtUBQ5-qF: GACGCTTCATCTCGTCC; UBQ5-qR: GTAAACGTAGGTGAGTCC) as a control, and the qPCR was conducted using 2 × M5 Hiper SYBR Premix (MF787-01, Mei5 Biotech) on a QuantStudio 5 Real-Time PCR System.

### Pathogen infection

*Colletotrichum higginsianum* was cultured on potato dextrose agar (PDA, 200 g potato infusion after boiling, 20 g D-glucose, and 20 g agar powder per liter) at 25 °C in the dark for 14 d. The conidia were suspended in potato dextrose broth medium (6 g/L potato extract and 20 g/L dextrose) and adjusted to 4 × 10^5^ spores/mL for testing the resistance of *BFP1* OE and *CYS6* OE. 10 mL of spore suspension was sprayed onto the leaves of 7-day-old plants per pot (7 × 7 cm). Later, plants were covered with a dome for 7 d to facilitate infection before being collected for the determination of fungal biomass. The biomass of *C. higginsianum* was quantified by qPCR analysis of *ChTUB* and *AtUBQ5*.

### Statistical analysis

All statistical analyses in this study were performed using GraphPad Prism 8 software, and significant differences were assessed by Student's *t*-test. The sample size *n* represents the number of biological replicates. All data are presented as mean ± standard deviation (mean ± SD). (ns indicates no significant difference; **p < 0.01; ***p < 0.001;****p < 0.0001).

## Results

**Figure 1.**
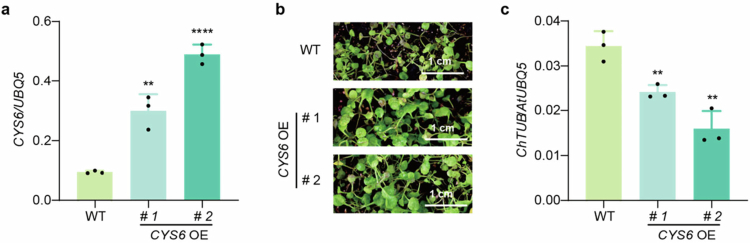
Overexpression of *CYS6* enhances plant resistances to *C. higginsianum*. (a) The expression levels of *CYS6* in their homozygous T_4_ transgenic lines. (b) Representative seedlings of 7-day-old WT and *CYS6* OE lines after sprayed with *C. higginsianum* (4 × 10^5^ spores/mL) for 7 d. (c) All of the infected seedlings of the same genotype are randomly collected into 3 biological samples to extract DNA for qPCR analysis of *ChTUB* with *AtUBQ5* as a control. Data are shown as means ± SD (*n* = 3 biological replicates for b). Significant differences compared with WT are detected using Student's *t*-test. **p < 0.01, ****p < 0.0001.

ROS are indispensable molecules in plant immunity.^12^ We previously reported that RBOHD, the key enzyme responsible for ROS production during immune responses, is degraded by xylem cysteine peptidase 1 (XCP1) in the vacuole under resting state to prevent inappropriate ROS accumulation.^16^ Upon pathogen infection, CYS6 could inhibit XCP1 activity, thereby stabilizing RBOHD and promoting ROS production. Thus, CYS6-XCP1-RBOHD module dynamically regulates ROS accumulation to balance growth and defense under different conditions. Consistently, *CYS6* overexpression (*CYS6* OE) lines exhibit elevated RBOHD levels and enhanced resistance to a broad spectrum of pathogens.^16^ In this study, we further investigated whether *CYS6* overexpression could enhance plant defense against *C. higginsianum*. Quantitative real-time PCR (qPCR) analysis confirmed that the expression level of *BFP1* was significantly upregulated compared with WT (Figure 1a). Seven-day-old seedlings of *CYS6* OE lines and wild-type (WT) plants were spray-inoculated with *C. higginsianum* spores. One-week later, WT plant displayed severe disease symptoms, whereas *CYS6* OE plants exhibited significantly reduced susceptibility (Figure 1b). To quantify pathogen growth, fungal biomass was assessed via qPCR using *ChTUB* gene as an indicator. As shown in Figure 1c, *CYS6* OE lines accumulated significantly less *C. higginsianum* biomass compared to WT, confirming the positive role of CYS6 in anthracnose resistance. Given that *CYS6* OE lines maintain higher RBOHD levels,^16^ we propose that CYS6 enhances resistance to *C. higginsianum* by sustaining elevated ROS production following pathogen infection.

**Figure 2.**
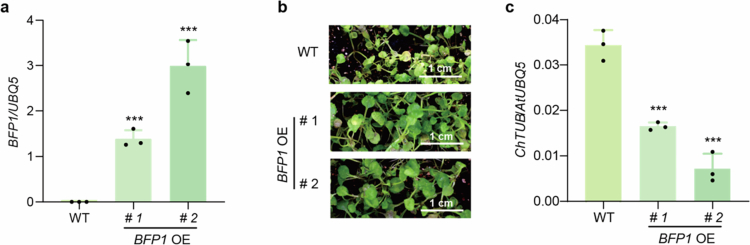
The *BFP1*-overexpressing lines shows insensitivity to *C. higginsianum* infection. (a) The expression levels of *BFP1* in their homozygous T_4_ transgenic lines. (b) Representative seedlings of 7-day-old WT and *BFP1* OE lines after sprayed with *C. higginsianum* (4 × 10^5^ spores/mL) for 7 d. (c) DNA is extracted from all infected seedlings for qPCR on *ChTUB* with *AtUBQ5* as a control. Data are shown as means ± SD (*n* = 3 biological replicates for b). Significant differences compared with WT are detected using Student's *t*-test. ***p < 0.001.

In addition to *CYS6*, we previously identified *BFP1* as another broad-spectrum resistance gene.^18^ *BFP1* encodes an F-box protein that is induced upon pathogen infection and promotes the degradation of JA-inactivating enzymes, the JAO (jasmonic acid oxidase) family proteins.^18^ In *BFP1* overexpression (*BFP1* OE) lines, JA levels are elevated during the early stages of pathogen infection. To determine whether BFP1 also enhances resistance to *C. higginsianum*, we verified by qPCR that *BFP1* expression was significantly increased relative to WT (Figure 2a). Subsequently, both WT and *BFP1* OE plants were inoculated with spores of *C. higginsianum*. As shown in Figure 2b and c, *BFP1* overexpression significantly reduced disease symptoms and pathogen accumulation compared to WT. We propose that BFP1 enhances plant defense against *C. higginsianum* by promoting JA accumulation.

**Figure 3.**
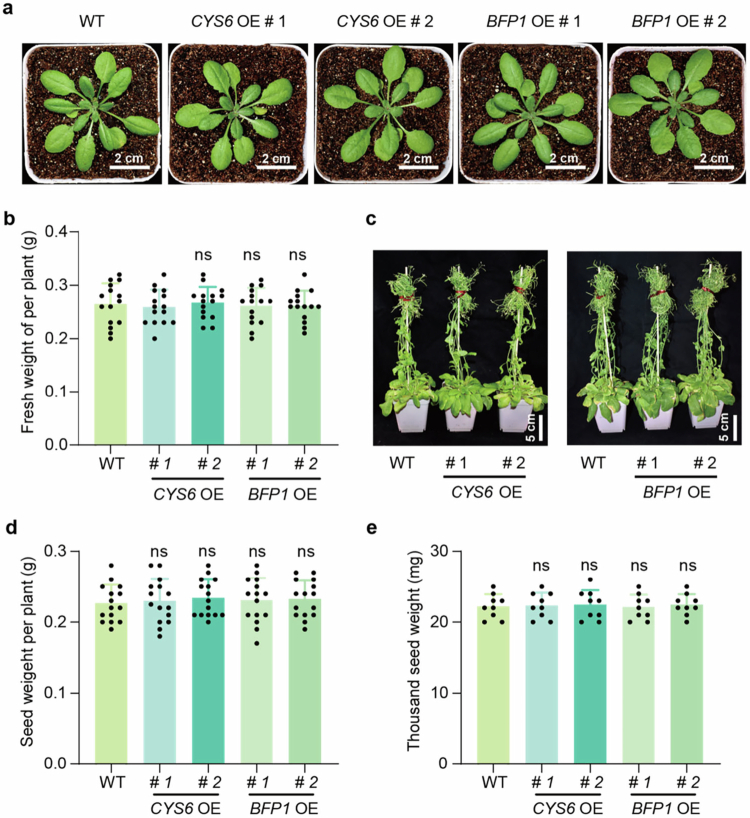
No growth defect is observed in *CYS6* OE and *BFP1* OE lines. (a and b) Representative phenotypes of 5-week-old WT, *CYS6* OE and *BFP1* OE transgenic lines are displayed and the fresh weight per plant is measured. (c) Representative plants of eight-week-old WT, *CYS6* OE, and *BFP1* OE plants. (d and e) The seed weight per plants and thousand seed weight of WT, *CYS6* OE, and *BFP1* OE lines. Data are shown as means ± SD (*n* = 15 biological replicates for b and d; *n* = 9 biological replicates for e). Significant differences compared with WT are detected using Student's *t*-test. ns, no significant difference.

Enhancing plant defense is generally correlated with growth defects, a phenomenon known as growth-defense tradeoff. Previously, we demonstrated that overexpressing *BFP1* has no effect on plant growth in the absence of pathogen infection.^18^ To determine whether CYS6 could similarly enhance defense without growth penalties like BFP1, we cultivated WT, *CYS6* OE, and *BFP1* OE lines in soil and monitored their growth phenotypes at 5 or 8 weeks after planting. As shown in Figure 3a–c, no significant differences were observed among these plants in terms of plant size, weight, or height. We further analyzed seed production by measuring the seed weight per plant. The average seed weights were 0.227 g/plant, 0.230 g/plant, 0.234 g/plant, 0.230 g/plant, 0.233 g/plant for WT, *CYS6* OE # 1, *CYS6* OE # 2, *BFP1* OE # 1, *BFP1* OE # 2, respectively, showing no significant variation among them (Figure 3d). In addition, no significant difference was observed between WT and either *CYS6* or *BFP1* OE lines in the weight of one thousand seeds (Figure 3e). These results collectively demonstrate that overexpressing *CYS6* and *BFP1* can enhance plant defense without incurring fitness costs.

**Figure 4.**
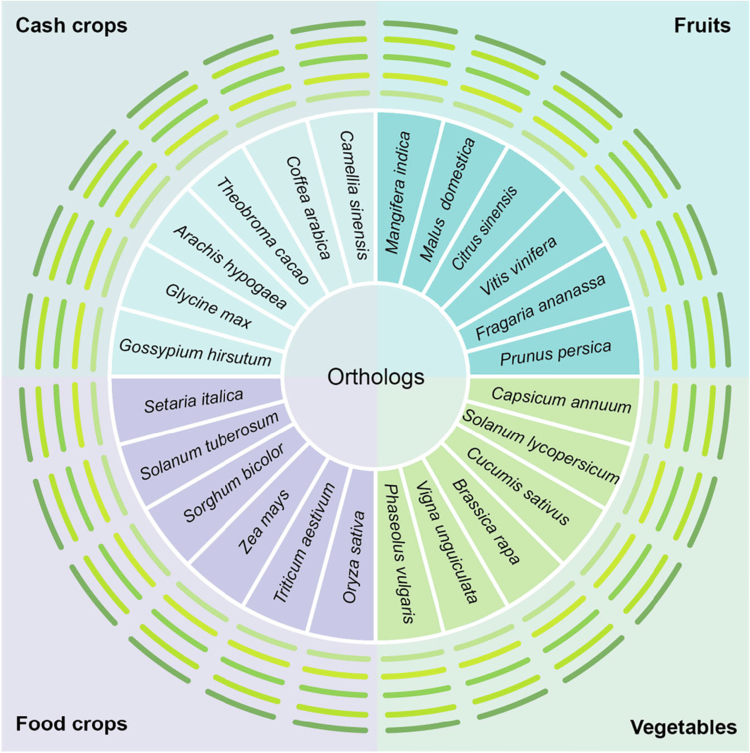
Presence of putative orthologs of CYS6, XCP1, RBOHD, BFP1, and JAO2 in different species. From inside to outside, the green short lines of varying shades represent CYS6, XCP1, RBOHD, BFP1, and JAO2, respectively, indicating the conservation levels of these genes across different species. These orthologs are acquired using NCBI-BLAST (https://blast.ncbi.nlm.nih.gov/Blast.cgi), TAIR (https://www.arabidopsis.org/) and the PLAZA (https://www.vandepoelelab.be/plaza/versions/plaza_v5_dicots/) database.

*Colletotrichum* spp. are severe pathogens affecting multiple crops, while ROS and JA are well-known molecules regulating plant defense in diverse plant species.^2-5^^,^^12^^,^^17^ We wonder whether the CYS6-XCP1-RBOHD module and BFP1-JAOs module are conserved among different crops. To explore this, we screened a broad range of 24 plant species, including food crops, cash crops, fruits, and vegetables, for the orthologs of CYS6, XCP1, RBOHD, BFP1, and the JAO family (represented by JAO2) to verify their evolutionary conservation (Additional file 1: Table 1–5 and Supplemental Figures 1–5). As shown in Figure 4, all components of both CYS6-XCP1-RBOHD and BFP1-JAOs modules displayed evolutionary conservation in the major crop plants we examined.

## Discussion

In *Arabidopsis*, ROS generation during PTI mainly relies on RBOHD, a membrane-localized protein containing six conserved transmembrane domains with its N- and C-termini located in the cytoplasm.^29^ Recognition of PAMPs by pattern recognition receptors (PRRs) triggers Ca^2+^ influx, which induces conformational alterations in the N-terminal EF-hand motifs of RBOHD and subsequent phosphorylation by CPKs, thereby leading to ROS burst.^30-34^ This early oxidative burst directly restricts pathogens at the local infection site and enhances systemic acquired resistance.^30^ Furthermore, ROS also activates the MAPK cascades and other late PTI responses,^35-37^ enhancing resistance to both biotrophic and necrotrophic pathogens.^12^^,^^38^ Nevertheless, excessive ROS accumulation is cytotoxic and may inhibit plant growth. Consequently, precise regulation of RBOHD protein abundance is necessary to avoid uncontrolled ROS burst and metabolic cost. Following phosphorylation by PBL13 and ubiquitination by PIRE at its C-terminus, RBOHD is transported to the vacuole,^39^^,^^40^ where it undergoes specific cleavage and degradation by XCP1.^16^

Previous research has established that CYS6 positively regulates Arabidopsis defense responses against *Pseudomonas syringae* pv. *maculicola* and *Botrytis cinerea*. In *cys6* mutants, PTI responses are significantly attenuated, manifested by reduced PAMP-induced phosphorylation of MPK3/6 and a diminished ROS burst. However, the expression of PAMP-induced PTI-related transcription factors, such as *WRKY29* and *WRKY33*, shows no significant difference compared with WT.^16^ These findings indicate that the CYS6-XCP1-RBOHD pathway specifically regulates the intensity of the ROS burst without affecting the transcriptional reprogramming downstream of PTI.

As a crucial defense phytohormone, the intracellular levels of JA are tightly regulated.^41^^,^^42^ The dynamic homeostasis of JA primarily depends on BFP1-mediated ubiquitination and degradation of JAOs. This process is specifically enhanced under pathogen stress to suppress JA catabolism, thereby maintaining high intracellular JA concentrations. Under non-stress conditions, *BFP1* is expressed at a basal level, ensuring appropriate turnover of JAOs to support normal plant growth and development.^18^ This regulatory module allows JA signaling to resolve the growth-defense antagonism and mount a rapid, effective immune response upon pathogen invasion. Together, the BFP1‑JAOs and CYS6‑XCP1‑RBOHD modules constitute a coordinated, broad‑spectrum disease resistance mechanism in plant immunity.

**Figure 5.**
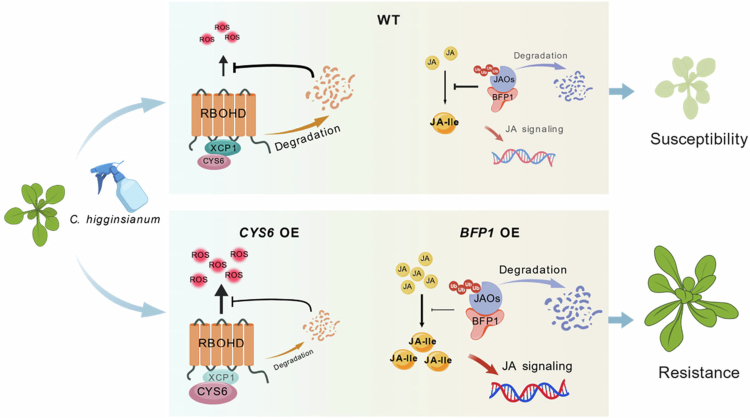
The working model of *CYS6* and *BFP1* overexpression enhances plant resistances to *C. higginsianum* without fitness costs. Upon *C. higginsianum* infection, XCP1-mediated degradation of RBOHD in WT caused a diminished ROS burst. Meanwhile, JAOs repressed JA-Ile biosynthesis, attenuating JA signaling activation and resulting in poor plant growth or death. In overexpression plants, CYS6 inhibited XCP1 activity, leading to RBOHD accumulation and a robust ROS burst; JAOs were degraded via ubiquitination, which abrogated JA catabolism, promoted JA-Ile accumulation, and enhanced JA-dependent defense responses. This working model was created using BioGDP (biogdp.com).

Building on prior findings and the present data, we have summarized the potential molecular mechanisms by which *CYS6* and *BFP1* overexpressing plants enhance resistance to anthracnose. In WT, the presence of CYS6 inhibits the function of XCP1, while BFP1 mediates the ubiquitination and degradation of JAOs. These actions maintain appropriate levels of RBOHD and JAOs, thereby ensuring proper intracellular ROS bursts and JA signaling responses.^16^^,^^18^ Compared to WT, the accumulation of higher amounts of CYS6 and BFP1 in overexpressing plants further restricts the functions of XCP1 and JAOs, enhances ROS bursts and JA signaling responses, and thus confers stronger anthracnose resistance to these overexpressing plants (Figure 5). Importantly, both CYS6 and BFP1 act as positive regulators of anthracnose resistance without imposing adaptive costs. We further showed that CYS6 and BFP1 regulatory functions are conserved across crops. These findings position *CYS6* and *BFP1* as promising candidates for breeding anthracnose-resistant crops.

## Supplementary Material

Supplementary Material Additional file 1.docx

## Data Availability

Not applicable.
