# Supplementary material for: Identifying anthracnose resistance genes involving in ROS and JA accumulation
Source: Plant Signal Behav. 2026 May 3;21(1):2668219. doi: 10.1080/15592324.2026.2668219 (PMC13138085; doi:10.1080/15592324.2026.2668219)
Supplement: Supplementary Material — Additional file 1.docx [file KPSB_A_2668219_SM0551.docx]

**Table 1 The orthologs of CYS6**

| **Crop categories** | **Species** | **The homologous protein with the highest sequence similarity to Arabidopsis** |
| --- | --- | --- |
| Fruits | *Mangifera indica* | LOC123193009 |
|  | *Malus domestica* | LOC103438579 |
|  | *Citrus sinensis* | LOC102618591 |
|  | *Vitis vinifera* | LOC100260839 |
|  | *Fragaria ananassa* | FAN01G0617 |
|  | *Prunus persica* | LOC18785448 |
| Vegetables | *Capsicum annuum* | LOC107862908 |
|  | *Solanum lycopersicum* | LOC543631 |
|  | *Cucumis sativus* | LOC101209225 |
|  | *Brassica rapa* | LOC103845535 |
|  | *Vigna unguiculata* | LOC114169761 |
|  | *Phaseolus vulgaris* | LOC137838653 |
| Food crops | *Oryza sativa* | LOC4326272 |
|  | *Triticum aestivum* | LOC123078150 |
|  | *Zea mays* | LOC732753 |
|  | *Sorghum bicolor* | LOC8054641 |
|  | *Solanum tuberosum* | LOC102591337 |
|  | *Setaria italica* | LOC101757583 |
| Cash crops | *Camellia sinensis* | LOC114301921 |
|  | *Coffea arabica* | LOC113729781 |
|  | *Theobroma cacao* | LOC18587059 |
|  | *Arachis hypogaea* | LOC112770680 |
|  | *Glycine max* | LOC547777 |
|  | *Gossypium hirsutum* | LOC121222495 |

**Table 2 The orthologs of XCP1**

| **Crop categories** | **Species** | **The homologous protein with the highest sequence similarity to Arabidopsis** |
| --- | --- | --- |
| Fruits | *Mangifera indica* | LOC123213986 |
|  | *Malus domestica* | LOC103445291 |
|  | *Citrus sinensis* | LOC102610106 |
|  | *Vitis vinifera* | LOC104877917 |
|  | *Fragaria ananassa* | FAN02G1139 |
|  | *Prunus persica* | LOC18766578 |
| Vegetables | *Capsicum annuum* | LOC107866767 |
|  | *Solanum lycopersicum* | LOC101266414 |
|  | *Cucumis sativus* | LOC101206340 |
|  | *Brassica rapa* | LOC103828815 |
|  | *Vigna unguiculata* | LOC114164061 |
|  | *Phaseolus vulgaris* | LOC137822029 |
| Food crops | *Oryza sativa* | LOC4337582 |
|  | *Triticum aestivum* | LOC123120442 |
|  | *Zea mays* | LOC103627649 |
|  | *Sorghum bicolor* | LOC8068658 |
|  | *Solanum tuberosum* | LOC102580817 |
|  | *Setaria italica* | LOC101779690 |
| Cash crops | *Camellia sinensis* | LOC114305840 |
|  | *Coffea arabica* | LOC113700355 |
|  | *Theobroma cacao* | LOC18608377 |
|  | *Arachis hypogaea* | LOC112705740 |
|  | *Glycine max* | LOC100788466 |
|  | *Gossypium hirsutum* | LOC107929366 |

**Table 3 The orthologs of RBOHD**

| **Crop categories** | **Species** | **The homologous protein with the highest sequence similarity to Arabidopsis** |
| --- | --- | --- |
| Fruits | *Mangifera indica* | LOC123226881 |
|  | *Malus domestica* | LOC103433139 |
|  | *Citrus sinensis* | LOC102616720 |
|  | *Vitis vinifera* | LOC100262614 |
|  | *Fragaria ananassa* | FAN07G0156 |
|  | *Prunus persica* | LOC18789695 |
| Vegetables | *Capsicum annuum* | LOC107862088 |
|  | *Solanum lycopersicum* | LOC543603 |
|  | *Cucumis sativus* | LOC101209614 |
|  | *Brassica rapa* | LOC103854509 |
|  | *Vigna unguiculata* | LOC114162979 |
|  | *Phaseolus vulgaris* | LOC137822832 |
| Food crops | *Oryza sativa* | LOC107278041 |
|  | *Triticum aestivum* | LOC123098889 |
|  | *Zea mays* | LOC100136880 |
|  | *Sorghum bicolor* | LOC8056984 |
|  | *Solanum tuberosum* | LOC102598898 |
|  | *Setaria italica* | LOC101766192 |
| Cash crops | *Camellia sinensis* | LOC114313244 |
|  | *Coffea arabica* | LOC113739669 |
|  | *Theobroma cacao* | LOC18591375 |
|  | *Arachis hypogaea* | LOC112695536 |
|  | *Glycine max* | LOC100820185 |
|  | *Gossypium hirsutum* | LOC107904951 |

**Table 4 The orthologs of BFP1**

| **Crop categories** | **Species** | **The homologous protein with the highest sequence similarity to Arabidopsis** |
| --- | --- | --- |
| Fruits | *Mangifera indica* | LOC123225026 |
|  | *Malus domestica* | LOC103436102 |
|  | *Citrus sinensis* | LOC102608632 |
|  | *Vitis vinifera* | LOC100244949 |
|  | *Fragaria ananassa* | FAN05G2004 |
|  | *Prunus persica* | LOC18773905 |
| Vegetables | *Capsicum annuum* | LOC107843697 |
|  | *Solanum lycopersicum* | LOC101266189 |
|  | *Cucumis sativus* | LOC101202909 |
|  | *Brassica rapa* | LOC103864776 |
|  | *Vigna unguiculata* | LOC114186254 |
|  | *Phaseolus vulgaris* | LOC137835615 |
| Food crops | *Oryza sativa* | LOC4335798 |
|  | *Triticum aestivum* | LOC123066020 |
|  | *Zea mays* | LOC103641623 |
|  | *Sorghum bicolor* | LOC8058655 |
|  | *Solanum tuberosum* | LOC102606243 |
|  | *Setaria italica* | LOC101755087 |
| Cash crops | *Camellia sinensis* | LOC114289071 |
|  | *Coffea arabica* | LOC113726312 |
|  | *Theobroma cacao* | LOC18590267 |
|  | *Arachis hypogaea* | LOC112706935 |
|  | *Glycine max* | LOC100781337 |
|  | *Gossypium hirsutum* | LOC107954125 |

**Table 5 The orthologs of JAO2**

| **Crop categories** | **Species** | **The homologous protein with the highest sequence similarity to Arabidopsis** |
| --- | --- | --- |
| Fruits | *Mangifera indica* | LOC123227076 |
|  | *Malus domestica* | LOC103408224 |
|  | *Citrus sinensis* | LOC102627583 |
|  | *Vitis vinifera* | LOC100245030 |
|  | *Fragaria x ananassa* | FAN02G2016 |
|  | *Prunus persica* | LOC18784896 |
| Vegetables | *Capsicum annuum* | LOC107845999 |
|  | *Solanum lycopersicum* | LOC101260801 |
|  | *Cucumis sativus* | LOC101204654 |
|  | *Brassica rapa* | LOC103855634 |
|  | *Vigna unguiculata* | LOC114195608 |
|  | *Phaseolus vulgaris* | LOC137825804 |
| Food crops | *Oryza sativa* | LOC4327401 |
|  | *Triticum aestivum* | LOC123062492 |
|  | *Zea mays* | LOC103629177 |
|  | *Sorghum bicolor* | LOC8071264 |
|  | *Solanum tuberosum* | LOC102603761 |
|  | *Setaria italica* | LOC101783997 |
| Cash crops | *Camellia sinensis* | LOC114287340 |
|  | *Coffea arabica* | LOC113730351 |
|  | *Theobroma cacao* | LOC18585844 |
|  | *Arachis hypogaea* | LOC112784840 |
|  | *Glycine max* | LOC100814471 |
|  | *Gossypium hirsutum* | LOC107960007 |

*
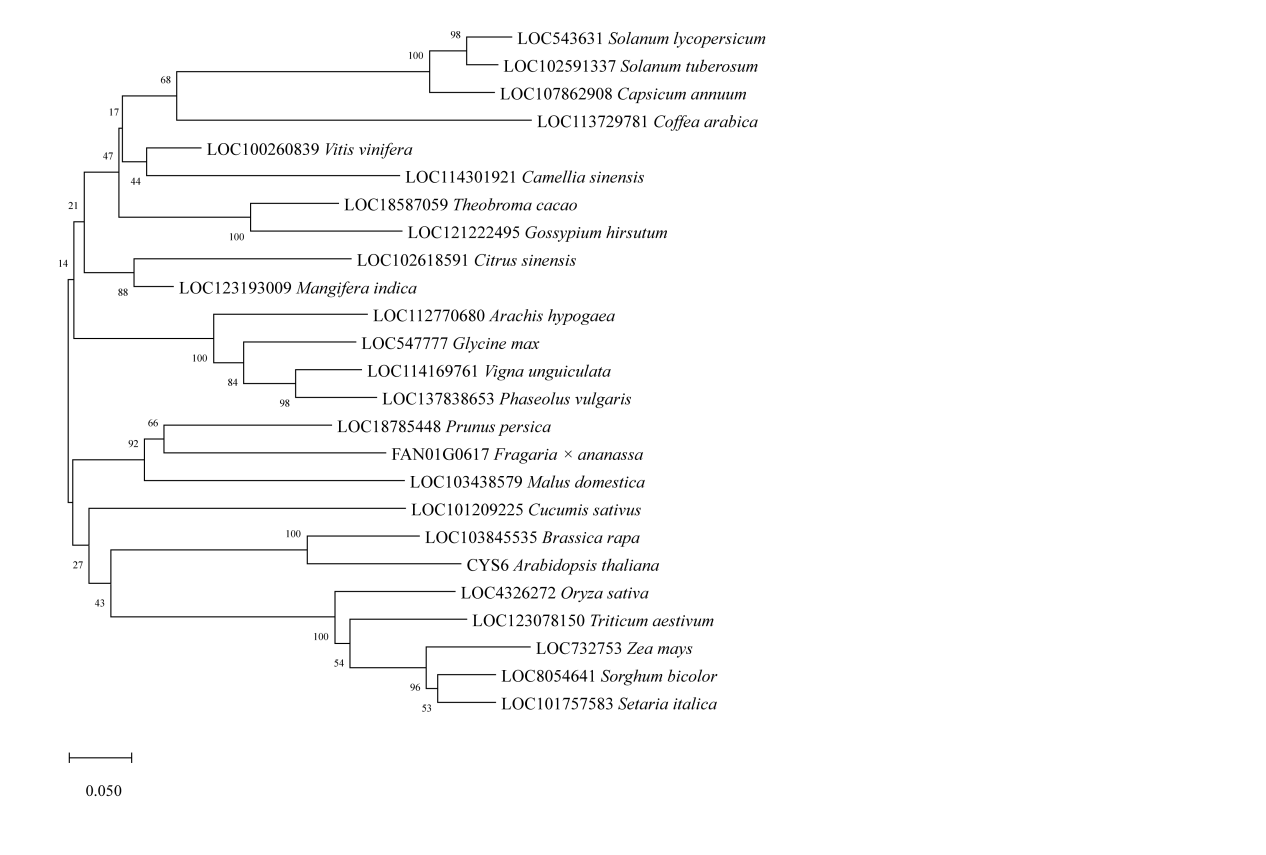
*

**Supplemental Figure 1 The phylogenetic tree based on protein sequences of AtCYS6.**

The protein sequences with the highest similarity to AtCYS6 in each species were selected and used to construct phylogenetic trees.Bootstrap values are shown in the tree. The scale represents branch length expressed as the relative number of amino acid substitutions.

**
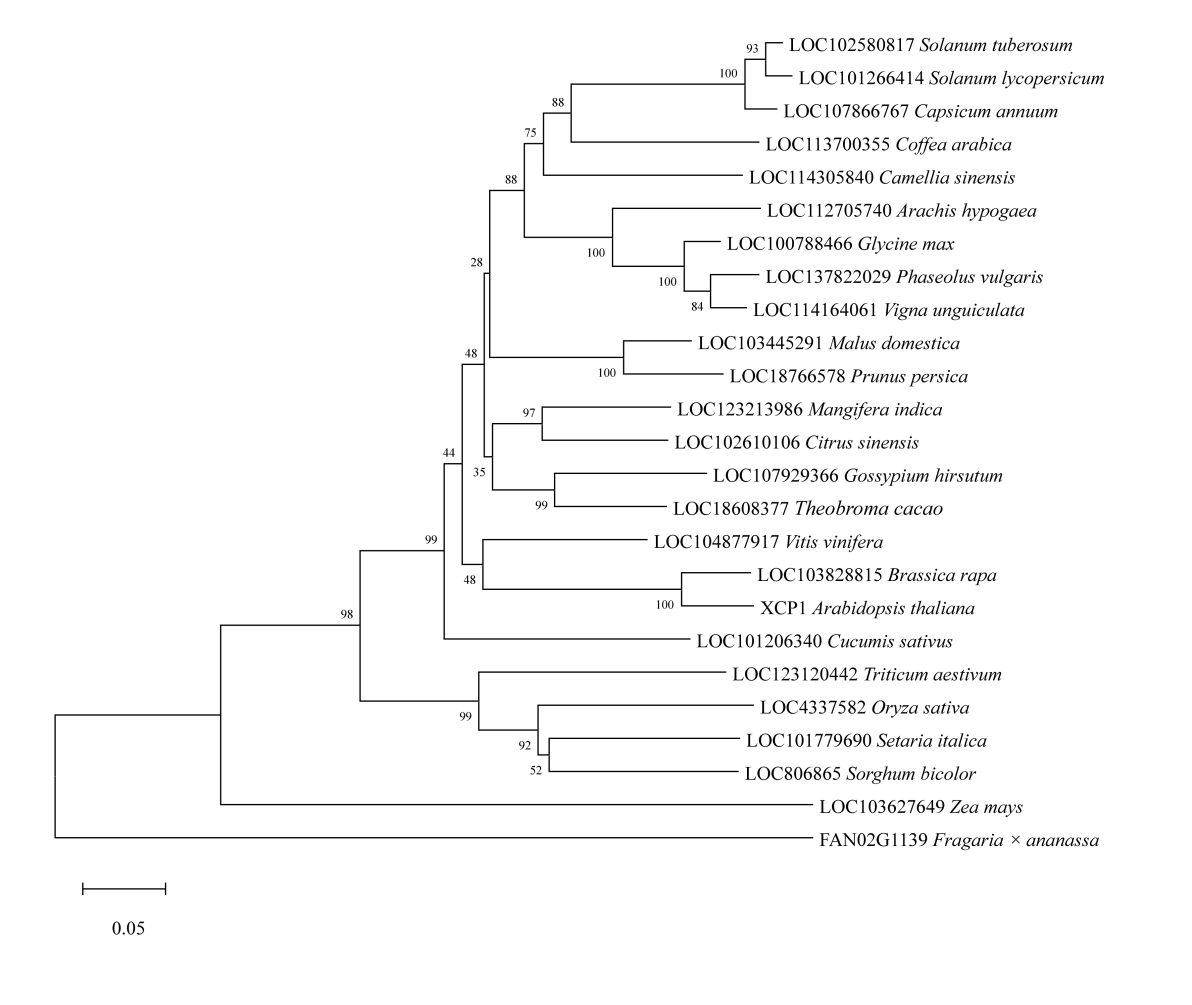
**

**Supplemental Figure 2 The phylogenetic tree based on protein sequences of AtXCP1.**

The protein sequences with the highest similarity to AtXCP1 in each species were selected and used to construct phylogenetic trees.Bootstrap values are shown in the tree. The scale represents branch length expressed as the relative number of amino acid substitutions.


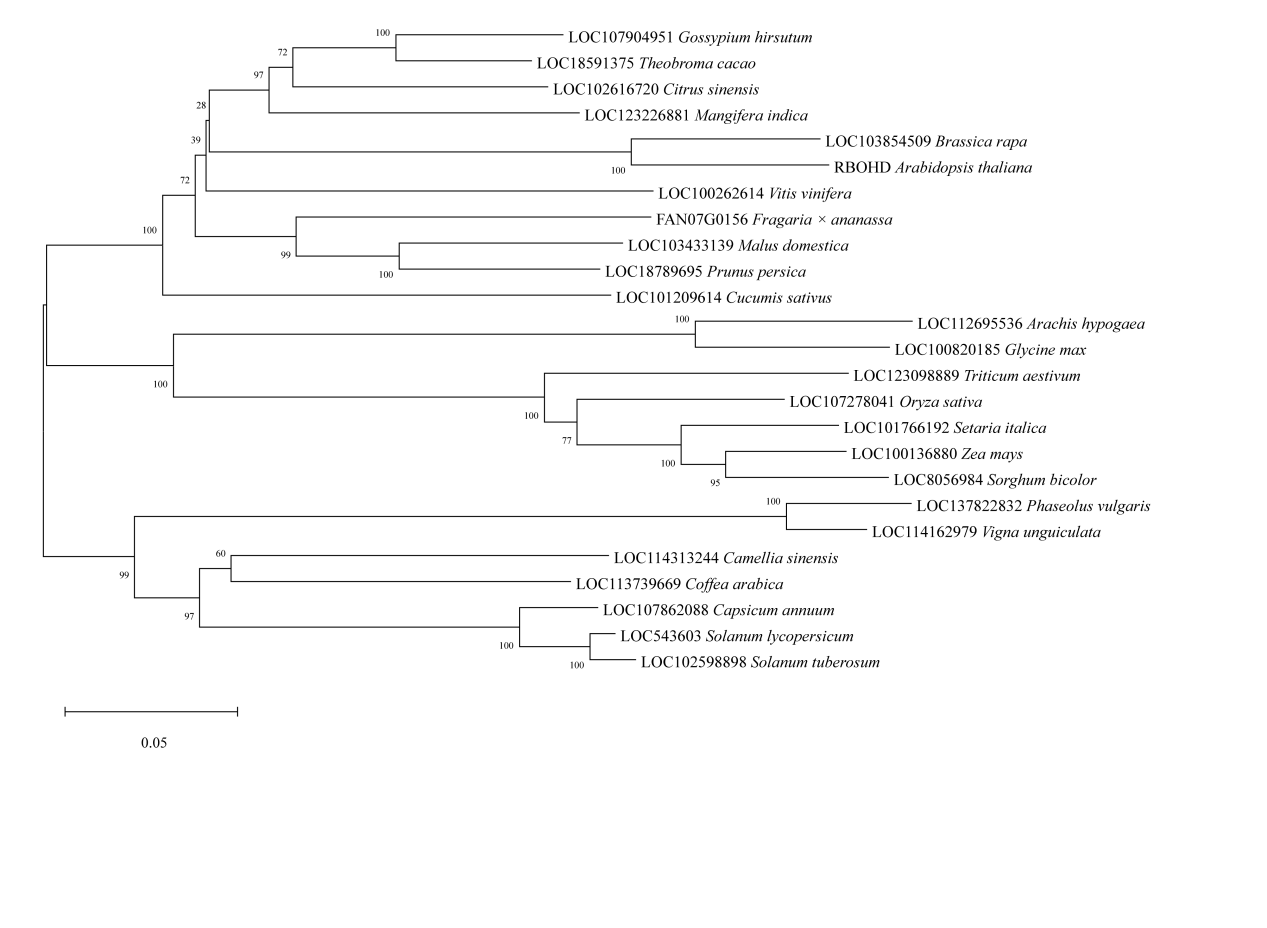


**Supplemental Figure 3 The phylogenetic tree based on protein sequences of AtRBOHD.**

The protein sequences with the highest similarity to AtRBOHD in each species were selected and used to construct phylogenetic trees.Bootstrap values are shown in the tree. The scale represents branch length expressed as the relative number of amino acid substitutions.


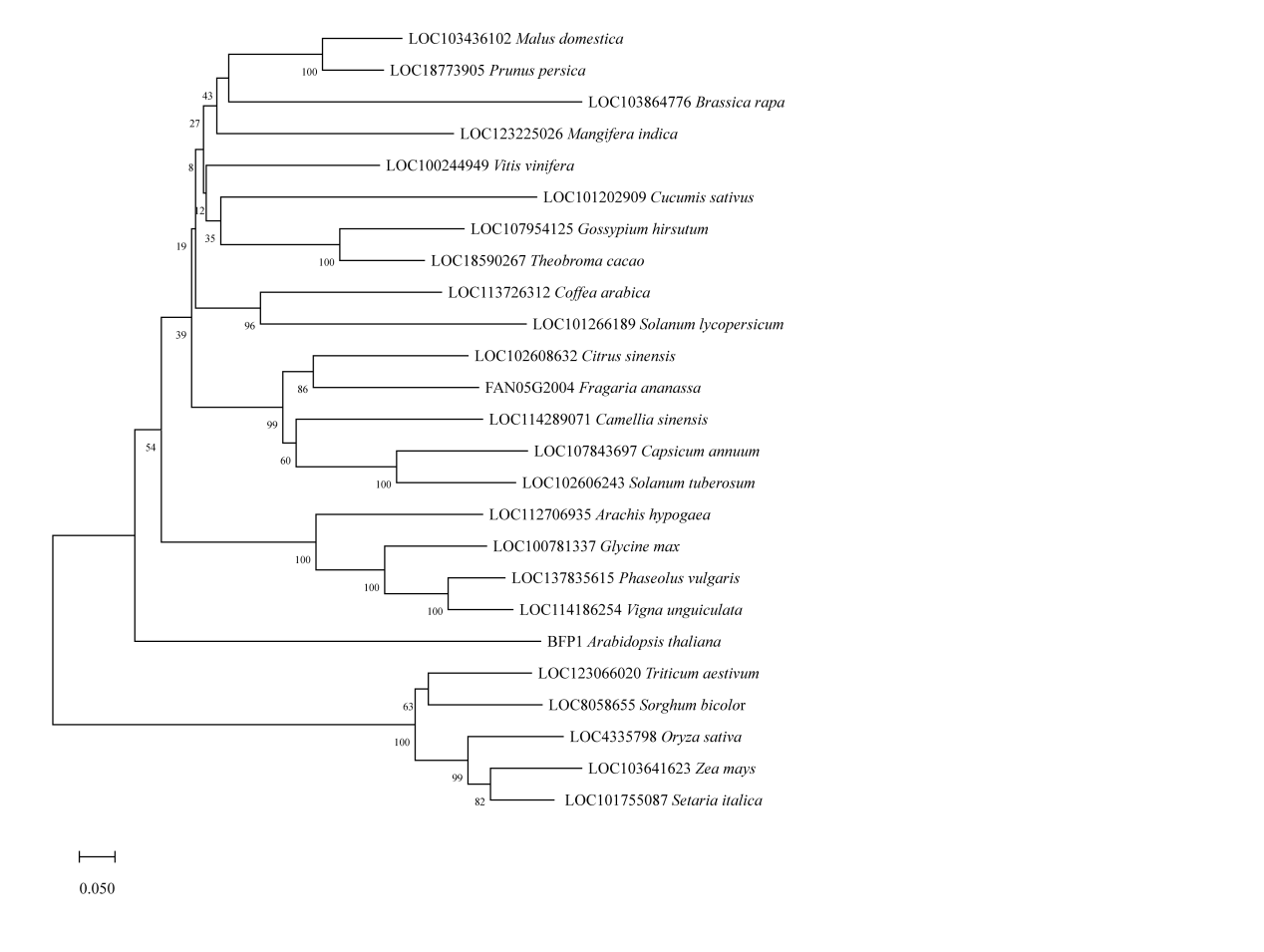


**Supplemental Figure 4 The phylogenetic tree based on protein sequences of AtBFP1.**

The protein sequences with the highest similarity to AtBFP1 in each species were selected and used to construct phylogenetic trees.Bootstrap values are shown in the tree. The scale represents branch length expressed as the relative number of amino acid substitutions.


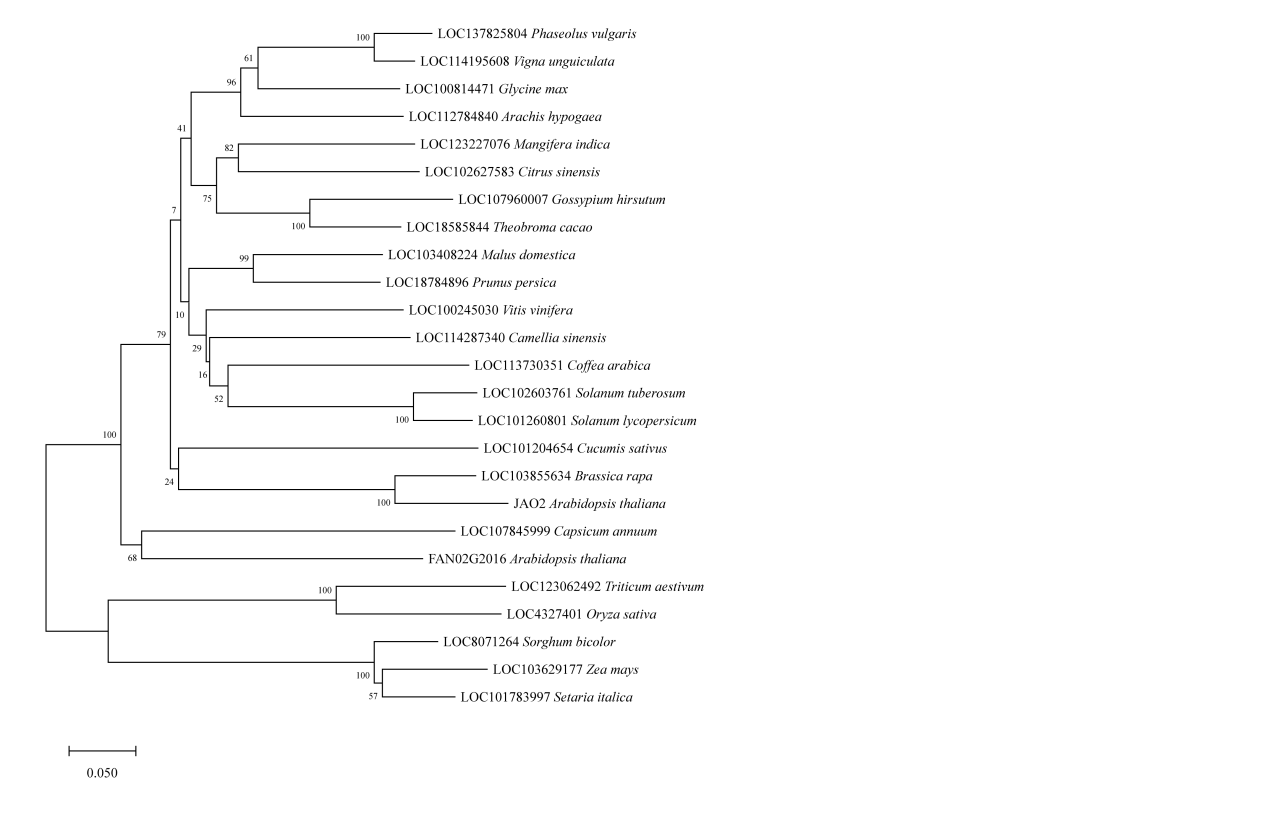


**Supplemental Figure 5 The phylogenetic tree based on protein sequences of AtJAO2.**

The protein sequences with the highest similarity to AtJAO2 in each species were selected and used to construct phylogenetic trees.Bootstrap values are shown in the tree. The scale represents branch length expressed as the relative number of amino acid substitutions.
